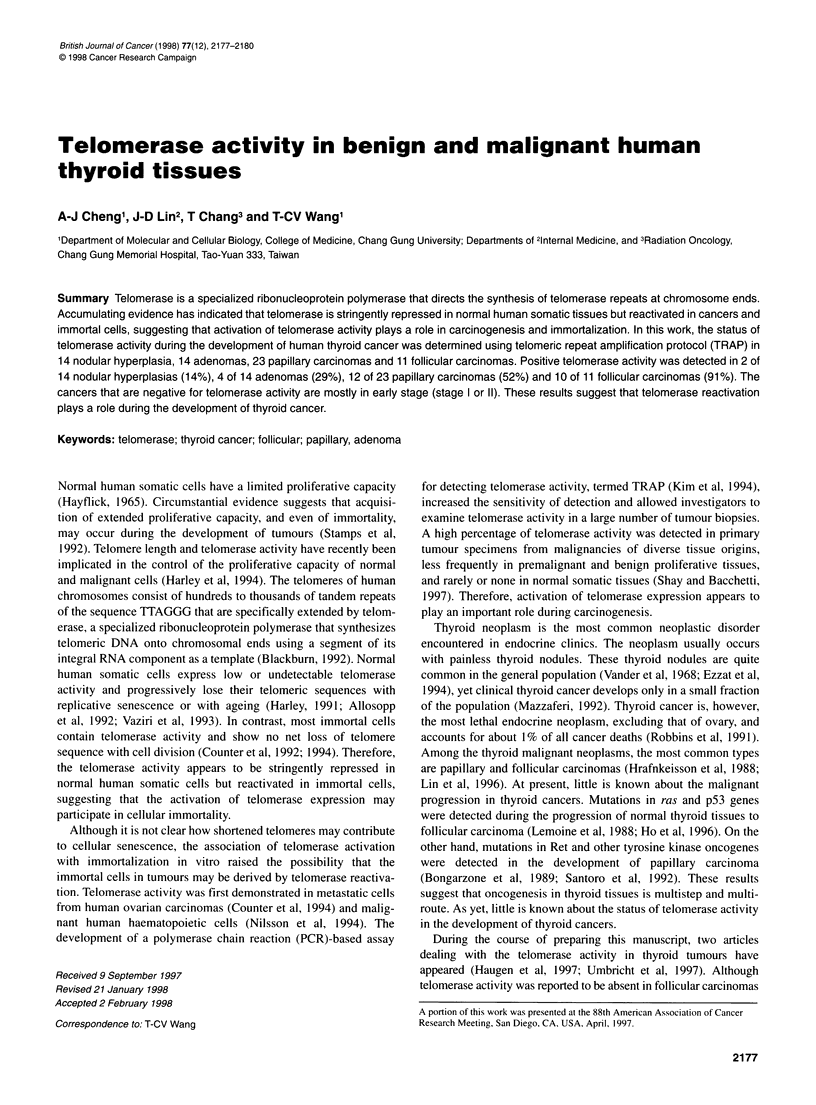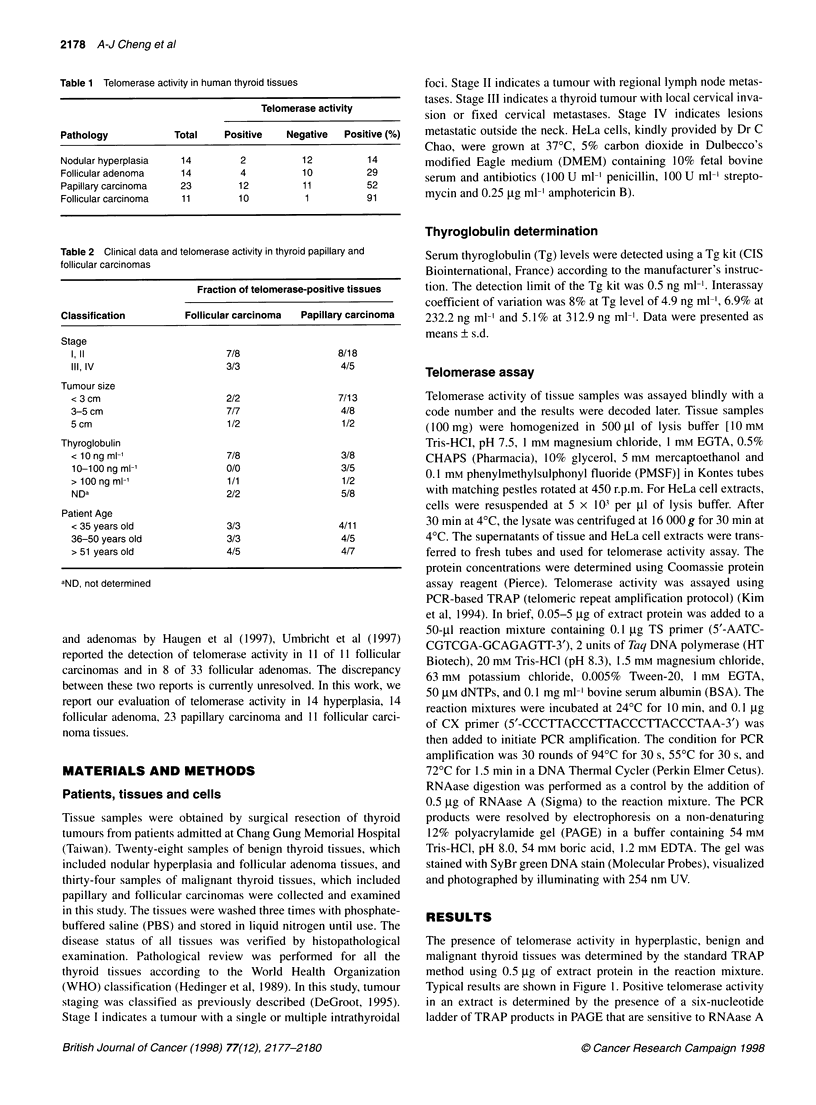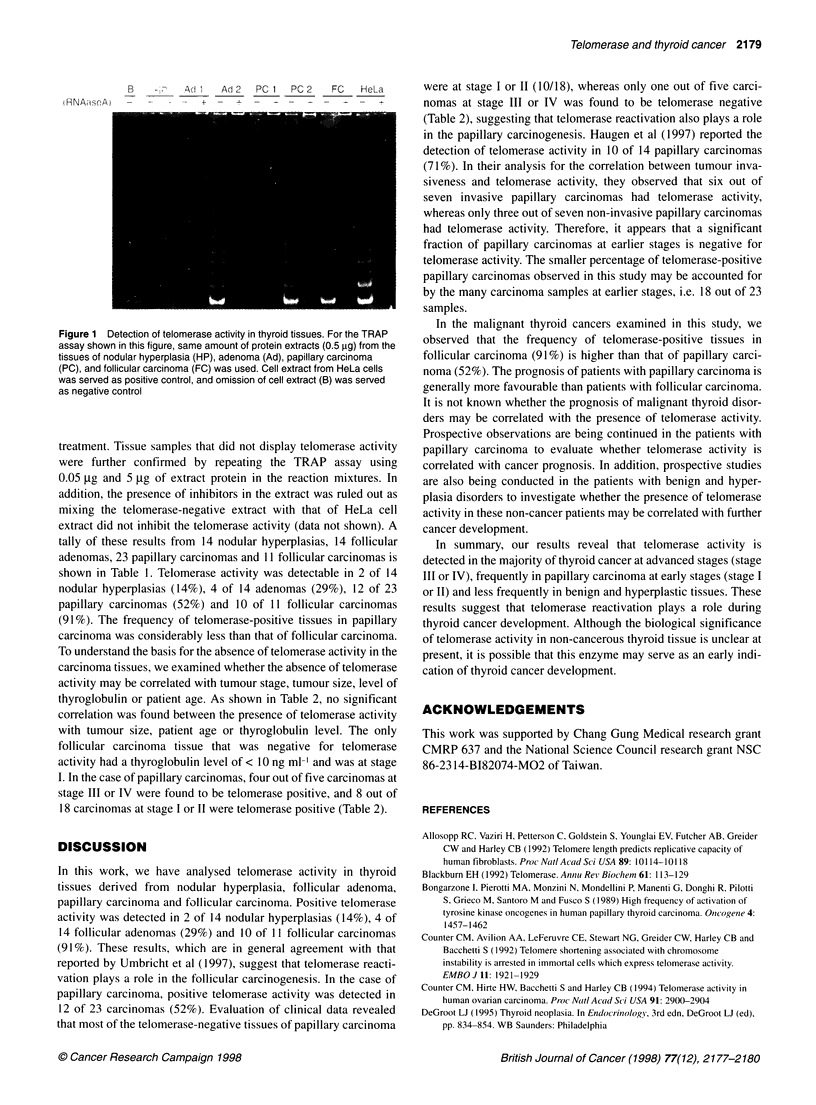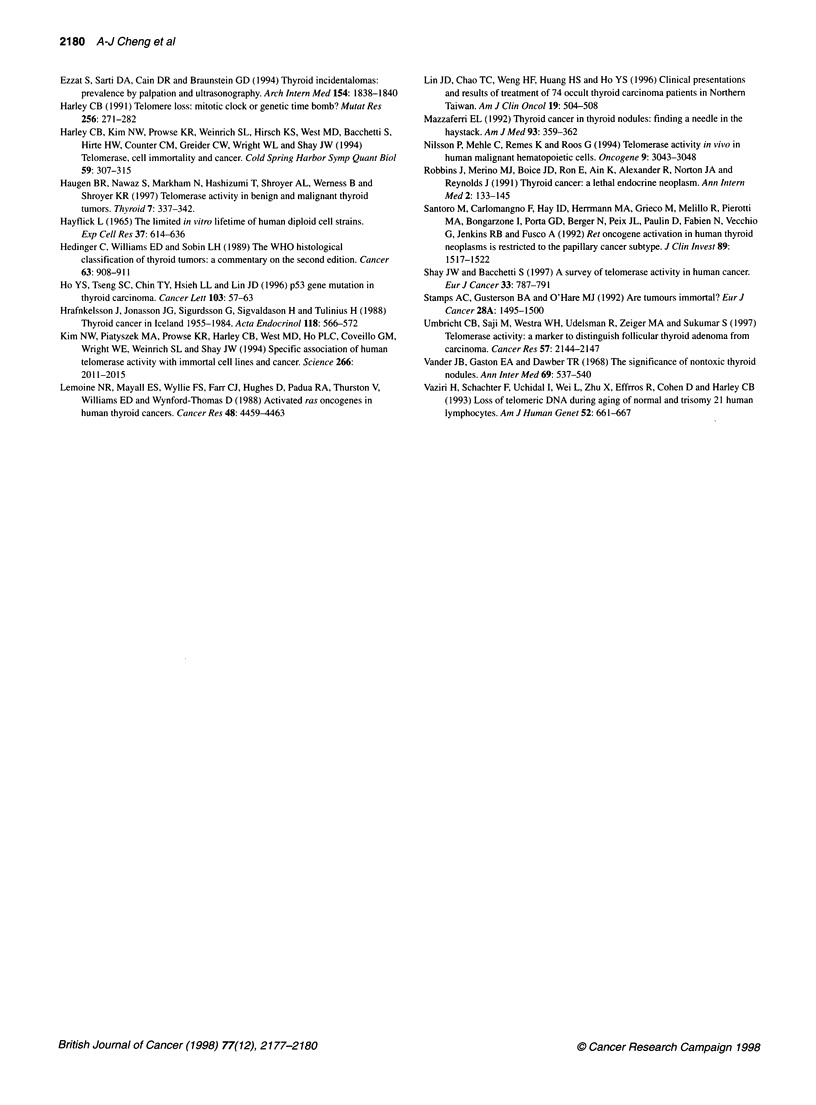# Telomerase activity in benign and malignant human thyroid tissues.

**DOI:** 10.1038/bjc.1998.363

**Published:** 1998-06

**Authors:** A. J. Cheng, J. D. Lin, T. Chang, T. C. Wang

**Affiliations:** Department of Molecular and Cellular Biology, College of Medicine, Chang Gung University, Tao-Yuan, Taiwan.

## Abstract

**Images:**


British Journal of Cancer (1998) 77(12), 2177–2180
© 1998 Cancer Research Campaign

### Telomerase activity in benign and malignant human thyroid tissues

**A-J Cheng[1], J-D Lin[2], T Chang[3] and T-CV Wang[1]**

[1]Department of Molecular and Cellular Biology, College of Medicine, Chang Gung University; Departments of [2]Internal Medicine, and [3]Radiation Oncology, Chang Gung Memorial Hospital, Tao-Yuan 333, Taiwan

**Summary** Telomerase is a specialized ribonucleoprotein polymerase that directs the synthesis of telomerase repeats at chromosome ends. Accumulating evidence has indicated that telomerase is stringently repressed in normal human somatic tissues but reactivated in cancers and immortal cells, suggesting that activation of telomerase activity plays a role in carcinogenesis and immortalization. In this work, the status of telomerase activity during the development of human thyroid cancer was determined using telomeric repeat amplification protocol (TRAP) in 14 nodular hyperplasia, 14 adenomas, 23 papillary carcinomas and 11 follicular carcinomas. Positive telomerase activity was detected in 2 of 14 nodular hyperplasias (14%), 4 of 14 adenomas (29%), 12 of 23 papillary carcinomas (52%) and 10 of 11 follicular carcinomas (91%). The cancers that are negative for telomerase activity are mostly in early stage (stage I or II). These results suggest that telomerase reactivation plays a role during the development of thyroid cancer.

**Keywords:** telomerase; thyroid cancer; follicular; papillary, adenoma

Normal human somatic cells have a limited proliferative capacity (Hayflick, 1965). Circumstantial evidence suggests that acquisition of extended proliferative capacity, and even of immortality, may occur during the development of tumours (Stamps et al, 1992). Telomere length and telomerase activity have recently been implicated in the control of the proliferative capacity of normal and malignant cells (Harley et al, 1994). The telomeres of human chromosomes consist of hundreds to thousands of tandem repeats of the sequence TTAGGG that are specifically extended by telomerase, a specialized ribonucleoprotein polymerase that synthesizes telomeric DNA onto chromosomal ends using a segment of its integral RNA component as a template (Blackburn, 1992). Normal human somatic cells express low or undetectable telomerase activity and progressively lose their telomeric sequences with replicative senescence or with ageing (Harley, 1991; Allosopp et al, 1992; Vaziri et al, 1993). In contrast, most immortal cells contain telomerase activity and show no net loss of telomere sequence with cell division (Counter et al, 1992; 1994). Therefore, the telomerase activity appears to be stringently repressed in normal human somatic cells but reactivated in immortal cells, suggesting that the activation of telomerase expression may participate in cellular immortality.

Although it is not clear how shortened telomeres may contribute to cellular senescence, the association of telomerase activation with immortalization in vitro raised the possibility that the immortal cells in tumours may be derived by telomerase reactivation. Telomerase activity was first demonstrated in metastatic cells from human ovarian carcinomas (Counter et al, 1994) and malignant human haematopoietic cells (Nilsson et al, 1994). The development of a polymerase chain reaction (PCR)-based assay for detecting telomerase activity, termed TRAP (Kim et al, 1994), increased the sensitivity of detection and allowed investigators to examine telomerase activity in a large number of tumour biopsies. A high percentage of telomerase activity was detected in primary tumour specimens from malignancies of diverse tissue origins, less frequently in premalignant and benign proliferative tissues, and rarely or none in normal somatic tissues (Shay and Bacchetti, 1997). Therefore, activation of telomerase expression appears to play an important role during carcinogenesis.

Thyroid neoplasm is the most common neoplastic disorder encountered in endocrine clinics. The neoplasm usually occurs with painless thyroid nodules. These thyroid nodules are quite common in the general population (Vander et al, 1968; Ezzat et al, 1994), yet clinical thyroid cancer develops only in a small fraction of the population (Mazzaferi, 1992). Thyroid cancer is, however, the most lethal endocrine neoplasm, excluding that of ovary, and accounts for about 1% of all cancer deaths (Robbins et al, 1991). Among the thyroid malignant neoplasms, the most common types are papillary and follicular carcinomas (Hrafnkeisson et al, 1988; Lin et al, 1996). At present, little is known about the malignant progression in thyroid cancers. Mutations in *ras* and p53 genes were detected during the progression of normal thyroid tissues to follicular carcinoma (Lemoine et al, 1988; Ho et al, 1996). On the other hand, mutations in Ret and other tyrosine kinase oncogenes were detected in the development of papillary carcinoma (Bongarzone et al, 1989; Santoro et al, 1992). These results suggest that oncogenesis in thyroid tissues is multistep and multiroute. As yet, little is known about the status of telomerase activity in the development of thyroid cancers.

During the course of preparing this manuscript, two articles dealing with the telomerase activity in thyroid tumours have appeared (Haugen et al, 1997; Umbricht et al, 1997). Although telomerase activity was reported to be absent in follicular carcinomas

*Received 9 September 1997*
*Revised 21 January 1998*
*Accepted 2 February 1998*

*Correspondence to:* T-CV Wang

A portion of this work was presented at the 88th American Association of Cancer Research Meeting, San Diego, CA, USA, April, 1997.

**Table 1** Telomerase activity in human thyroid tissues

| Pathology | Total | Telomerase activity | | |
|---|---|---|---|---|
| | | Positive | Negative | Positive (%) |
| Nodular hyperplasia | 14 | 2 | 12 | 14 |
| Follicular adenoma | 14 | 4 | 10 | 29 |
| Papillary carcinoma | 23 | 12 | 11 | 52 |
| Follicular carcinoma | 11 | 10 | 1 | 91 |

**Table 2** Clinical data and telomerase activity in thyroid papillary and follicular carcinomas

| Classification | Fraction of telomerase-positive tissues | |
|---|---|---|
| | Follicular carcinoma | Papillary carcinoma |
| Stage | | |
| I, II | 7/8 | 8/18 |
| III, IV | 3/3 | 4/5 |
| Tumour size | | |
| < 3 cm | 2/2 | 7/13 |
| 3–5 cm | 7/7 | 4/8 |
| 5 cm | 1/2 | 1/2 |
| Thyroglobulin | | |
| < 10 ng $ml^{-1}$ | 7/8 | 3/8 |
| 10–100 ng $ml^{-1}$ | 0/0 | 3/5 |
| > 100 ng $ml^{-1}$ | 1/1 | 1/2 |
| ND[a] | 2/2 | 5/8 |
| Patient Age | | |
| < 35 years old | 3/3 | 4/11 |
| 36–50 years old | 3/3 | 4/5 |
| > 51 years old | 4/5 | 4/7 |

[a]ND, not determined and adenomas by Haugen et al (1997), Umbricht et al (1997) reported the detection of telomerase activity in 11 of 11 follicular carcinomas and in 8 of 33 follicular adenomas. The discrepancy between these two reports is currently unresolved. In this work, we report our evaluation of telomerase activity in 14 hyperplasia, 14 follicular adenoma, 23 papillary carcinoma and 11 follicular carcinoma tissues.

### MATERIALS AND METHODS

### Patients, tissues and cells

Tissue samples were obtained by surgical resection of thyroid tumours from patients admitted at Chang Gung Memorial Hospital (Taiwan). Twenty-eight samples of benign thyroid tissues, which included nodular hyperplasia and follicular adenoma tissues, and thirty-four samples of malignant thyroid tissues, which included papillary and follicular carcinomas were collected and examined in this study. The tissues were washed three times with phosphate-buffered saline (PBS) and stored in liquid nitrogen until use. The disease status of all tissues was verified by histopathological examination. Pathological review was performed for all the thyroid tissues according to the World Health Organization (WHO) classification (Hedinger et al, 1989). In this study, tumour staging was classified as previously described (DeGroot, 1995). Stage I indicates a tumour with a single or multiple intrathyroidal foci. Stage II indicates a tumour with regional lymph node metastases. Stage III indicates a thyroid tumour with local cervical invasion or fixed cervical metastases. Stage IV indicates lesions metastatic outside the neck. HeLa cells, kindly provided by Dr C Chao, were grown at 37°C, 5% carbon dioxide in Dulbecco's modified Eagle medium (DMEM) containing 10% fetal bovine serum and antibiotics (100 U $ml^{-1}$ penicillin, 100 U $ml^{-1}$ streptomycin and 0.25 μg $ml^{-1}$ amphotericin B).

### Thyroglobulin determination

Serum thyroglobulin (Tg) levels were detected using a Tg kit (CIS Biointernational, France) according to the manufacturer's instruction. The detection limit of the Tg kit was 0.5 ng $ml^{-1}$. Interassay coefficient of variation was 8% at Tg level of 4.9 ng $ml^{-1}$, 6.9% at 232.2 ng $ml^{-1}$ and 5.1% at 312.9 ng $ml^{-1}$. Data were presented as means ± s.d.

### Telomerase assay

Telomerase activity of tissue samples was assayed blindly with a code number and the results were decoded later. Tissue samples (100 mg) were homogenized in 500 μl of lysis buffer [10 mM Tris-HCl, pH 7.5, 1 mM magnesium chloride, 1 mM EGTA, 0.5% CHAPS (Pharmacia), 10% glycerol, 5 mM mercaptoethanol and 0.1 mM phenylmethylsulphonyl fluoride (PMSF)] in Kontes tubes with matching pestles rotated at 450 r.p.m. For HeLa cell extracts, cells were resuspended at $5 \times 10^3$ per μl of lysis buffer. After 30 min at 4°C, the lysate was centrifuged at 16 000 ***g*** for 30 min at 4°C. The supernatants of tissue and HeLa cell extracts were transferred to fresh tubes and used for telomerase activity assay. The protein concentrations were determined using Coomassie protein assay reagent (Pierce). Telomerase activity was assayed using PCR-based TRAP (telomeric repeat amplification protocol) (Kim et al, 1994). In brief, 0.05–5 μg of extract protein was added to a 50-μl reaction mixture containing 0.1 μg TS primer (5′-AATC-CGTCGA-GCAGAGTT-3′), 2 units of *Taq* DNA polymerase (HT Biotech), 20 mM Tris-HCl (pH 8.3), 1.5 mM magnesium chloride, 63 mM potassium chloride, 0.005% Tween-20, 1 mM EGTA, 50 μM dNTPs, and 0.1 mg $ml^{-1}$ bovine serum albumin (BSA). The reaction mixtures were incubated at 24°C for 10 min, and 0.1 μg of CX primer (5′-CCCTTACCCTTACCCTTACCCTAA-3′) was then added to initiate PCR amplification. The condition for PCR amplification was 30 rounds of 94°C for 30 s, 55°C for 30 s, and 72°C for 1.5 min in a DNA Thermal Cycler (Perkin Elmer Cetus). RNAase digestion was performed as a control by the addition of 0.5 μg of RNAase A (Sigma) to the reaction mixture. The PCR products were resolved by electrophoresis on a non-denaturing 12% polyacrylamide gel (PAGE) in a buffer containing 54 mM Tris-HCl, pH 8.0, 54 mM boric acid, 1.2 mM EDTA. The gel was stained with SyBr green DNA stain (Molecular Probes), visualized and photographed by illuminating with 254 nm UV.

### RESULTS

The presence of telomerase activity in hyperplastic, benign and malignant thyroid tissues was determined by the standard TRAP method using 0.5 μg of extract protein in the reaction mixture. Typical results are shown in Figure 1. Positive telomerase activity in an extract is determined by the presence of a six-nucleotide ladder of TRAP products in PAGE that are sensitive to RNAase A

© Cancer Research Campaign 1998

**Figure 1** Detection of telomerase activity in thyroid tissues. For the TRAP assay shown in this figure, same amount of protein extracts (0.5 µg) from the tissues of nodular hyperplasia (HP), adenoma (Ad), papillary carcinoma (PC), and follicular carcinoma (FC) was used. Cell extract from HeLa cells was served as positive control, and omission of cell extract (B) was served as negative control treatment. Tissue samples that did not display telomerase activity were further confirmed by repeating the TRAP assay using 0.05 µg and 5 µg of extract protein in the reaction mixtures. In addition, the presence of inhibitors in the extract was ruled out as mixing the telomerase-negative extract with that of HeLa cell extract did not inhibit the telomerase activity (data not shown). A tally of these results from 14 nodular hyperplasias, 14 follicular adenomas, 23 papillary carcinomas and 11 follicular carcinomas is shown in Table 1. Telomerase activity was detectable in 2 of 14 nodular hyperplasias (14%), 4 of 14 adenomas (29%), 12 of 23 papillary carcinomas (52%) and 10 of 11 follicular carcinomas (91%). The frequency of telomerase-positive tissues in papillary carcinoma was considerably less than that of follicular carcinoma. To understand the basis for the absence of telomerase activity in the carcinoma tissues, we examined whether the absence of telomerase activity may be correlated with tumour stage, tumour size, level of thyroglobulin or patient age. As shown in Table 2, no significant correlation was found between the presence of telomerase activity with tumour size, patient age or thyroglobulin level. The only follicular carcinoma tissue that was negative for telomerase activity had a thyroglobulin level of < 10 ng $ml^{-1}$ and was at stage I. In the case of papillary carcinomas, four out of five carcinomas at stage III or IV were found to be telomerase positive, and 8 out of 18 carcinomas at stage I or II were telomerase positive (Table 2).

### DISCUSSION

In this work, we have analysed telomerase activity in thyroid tissues derived from nodular hyperplasia, follicular adenoma, papillary carcinoma and follicular carcinoma. Positive telomerase activity was detected in 2 of 14 nodular hyperplasias (14%), 4 of 14 follicular adenomas (29%) and 10 of 11 follicular carcinomas (91%). These results, which are in general agreement with that reported by Umbricht et al (1997), suggest that telomerase reactivation plays a role in the follicular carcinogenesis. In the case of papillary carcinoma, positive telomerase activity was detected in 12 of 23 carcinomas (52%). Evaluation of clinical data revealed that most of the telomerase-negative tissues of papillary carcinoma were at stage I or II (10/18), whereas only one out of five carcinomas at stage III or IV was found to be telomerase negative (Table 2), suggesting that telomerase reactivation also plays a role in the papillary carcinogenesis. Haugen et al (1997) reported the detection of telomerase activity in 10 of 14 papillary carcinomas (71%). In their analysis for the correlation between tumour invasiveness and telomerase activity, they observed that six out of seven invasive papillary carcinomas had telomerase activity, whereas only three out of seven non-invasive papillary carcinomas had telomerase activity. Therefore, it appears that a significant fraction of papillary carcinomas at earlier stages is negative for telomerase activity. The smaller percentage of telomerase-positive papillary carcinomas observed in this study may be accounted for by the many carcinoma samples at earlier stages, i.e. 18 out of 23 samples.

In the malignant thyroid cancers examined in this study, we observed that the frequency of telomerase-positive tissues in follicular carcinoma (91%) is higher than that of papillary carcinoma (52%). The prognosis of patients with papillary carcinoma is generally more favourable than patients with follicular carcinoma. It is not known whether the prognosis of malignant thyroid disorders may be correlated with the presence of telomerase activity. Prospective observations are being continued in the patients with papillary carcinoma to evaluate whether telomerase activity is correlated with cancer prognosis. In addition, prospective studies are also being conducted in the patients with benign and hyperplasia disorders to investigate whether the presence of telomerase activity in these non-cancer patients may be correlated with further cancer development.

In summary, our results reveal that telomerase activity is detected in the majority of thyroid cancer at advanced stages (stage III or IV), frequently in papillary carcinoma at early stages (stage I or II) and less frequently in benign and hyperplastic tissues. These results suggest that telomerase reactivation plays a role during thyroid cancer development. Although the biological significance of telomerase activity in non-cancerous thyroid tissue is unclear at present, it is possible that this enzyme may serve as an early indication of thyroid cancer development.

### ACKNOWLEDGEMENTS

This work was supported by Chang Gung Medical research grant CMRP 637 and the National Science Council research grant NSC 86-2314-BI82074-MO2 of Taiwan.

### REFERENCES

Allosopp RC, Vaziri H, Petterson C, Goldstein S, Younglai EV, Futcher AB, Greider CW and Harley CB (1992) Telomere length predicts replicative capacity of human fibroblasts. *Proc Natl Acad Sci USA* **89**: 10114–10118

Blackburn EH (1992) Telomerase. *Annu Rev Biochem* **61**: 113–129

Bongarzone I, Pierotti MA, Monzini N, Mondellini P, Manenti G, Donghi R, Pilotti S, Grieco M, Santoro M and Fusco S (1989) High frequency of activation of tyrosine kinase oncogenes in human papillary thyroid carcinoma. *Oncogene* **4**: 1457–1462

Counter CM, Avilion AA, LeFeruvre CE, Stewart NG, Greider CW, Harley CB and Bacchetti S (1992) Telomere shortening associated with chromosome instability is arrested in immortal cells which express telomerase activity. *EMBO J* **11**: 1921–1929

Counter CM, Hirte HW, Bacchetti S and Harley CB (1994) Telomerase activity in human ovarian carcinoma. *Proc Natl Acad Sci USA* **91**: 2900–2904

DeGroot LJ (1995) Thyroid neoplasia. In *Endocrinology*, 3rd edn, DeGroot LJ (ed), pp. 834–854. WB Saunders: Philadelphia

© Cancer Research Campaign 1998 

Ezzat S, Sarti DA, Cain DR and Braunstein GD (1994) Thyroid incidentalomas: prevalence by palpation and ultrasonography. *Arch Intern Med* **154**: 1838–1840

Harley CB (1991) Telomere loss: mitotic clock or genetic time bomb? *Mutat Res* **256**: 271–282

Harley CB, Kim NW, Prowse KR, Weinrich SL, Hirsch KS, West MD, Bacchetti S, Hirte HW, Counter CM, Greider CW, Wright WL and Shay JW (1994) Telomerase, cell immortality and cancer. *Cold Spring Harbor Symp Quant Biol* **59**: 307–315

Haugen BR, Nawaz S, Markham N, Hashizumi T, Shroyer AL, Werness B and Shroyer KR (1997) Telomerase activity in benign and malignant thyroid tumors. *Thyroid* **7**: 337–342.

Hayflick L (1965) The limited *in vitro* lifetime of human diploid cell strains. *Exp Cell Res* **37**: 614–636

Hedinger C, Williams ED and Sobin LH (1989) The WHO histological classification of thyroid tumors: a commentary on the second edition. *Cancer* **63**: 908–911

Ho YS, Tseng SC, Chin TY, Hsieh LL and Lin JD (1996) p53 gene mutation in thyroid carcinoma. *Cancer Lett* **103**: 57–63

Hrafnkelsson J, Jonasson JG, Sigurdsson G, Sigvaldason H and Tulinius H (1988) Thyroid cancer in Iceland 1955–1984. *Acta Endocrinol* **118**: 566–572

Kim NW, Piatyszek MA, Prowse KR, Harley CB, West MD, Ho PLC, Coveillo GM, Wright WE, Weinrich SL and Shay JW (1994) Specific association of human telomerase activity with immortal cell lines and cancer. *Science* **266**: 2011–2015

Lemoine NR, Mayall ES, Wyllie FS, Farr CJ, Hughes D, Padua RA, Thurston V, Williams ED and Wynford-Thomas D (1988) Activated *ras* oncogenes in human thyroid cancers. *Cancer Res* **48**: 4459–4463

Lin JD, Chao TC, Weng HF, Huang HS and Ho YS (1996) Clinical presentations and results of treatment of 74 occult thyroid carcinoma patients in Northern Taiwan. *Am J Clin Oncol* **19**: 504–508

Mazzaferri EL (1992) Thyroid cancer in thyroid nodules: finding a needle in the haystack. *Am J Med* **93**: 359–362

Nilsson P, Mehle C, Remes K and Roos G (1994) Telomerase activity *in vivo* in human malignant hematopoietic cells. *Oncogene* **9**: 3043–3048

Robbins J, Merino MJ, Boice JD, Ron E, Ain K, Alexander R, Norton JA and Reynolds J (1991) Thyroid cancer: a lethal endocrine neoplasm. *Ann Intern Med* **2**: 133–145

Santoro M, Carlomangno F, Hay ID, Herrmann MA, Grieco M, Melillo R, Pierotti MA, Bongarzone I, Porta GD, Berger N, Peix JL, Paulin D, Fabien N, Vecchio G, Jenkins RB and Fusco A (1992) *Ret* oncogene activation in human thyroid neoplasms is restricted to the papillary cancer subtype. *J Clin Invest* **89**: 1517–1522

Shay JW and Bacchetti S (1997) A survey of telomerase activity in human cancer. *Eur J Cancer* **33**: 787–791

Stamps AC, Gusterson BA and O'Hare MJ (1992) Are tumours immortal? *Eur J Cancer* **28A**: 1495–1500

Umbricht CB, Saji M, Westra WH, Udelsman R, Zeiger MA and Sukumar S (1997) Telomerase activity: a marker to distinguish follicular thyroid adenoma from carcinoma. *Cancer Res* **57**: 2144–2147

Vander JB, Gaston EA and Dawber TR (1968) The significance of nontoxic thyroid nodules. *Ann Inter Med* **69**: 537–540

Vaziri H, Schachter F, Uchidal I, Wei L, Zhu X, Effrros R, Cohen D and Harley CB (1993) Loss of telomeric DNA during aging of normal and trisomy 21 human lymphocytes. *Am J Human Genet* **52**: 661–667

© Cancer Research Campaign 1998